# Chicago Medical Society

**Published:** 1869-07-15

**Authors:** Hiram Wanzer

**Affiliations:** Secretary


### SOCIETY REPORTS.

### *Chicago Medical Society.*

FRIDAY EVENING, *June* 11, 1869.

Regular meeting Chicago Medical Society, President Bogue in the chair. Minutes of last meeting were read and approved. Proposals for memberspip being in order, Dr. F. Bartels was recommended by Drs. Smith and Paoli, which was referred to the Board of Censors. Reports of Cases and Pathological Specimens being in order, Dr. Gray presented a specimen of a section of small intestine, and part of the colon, with its contiguous peritoneum. The Doctor stated the premonitory symptoms were dysentery, vomiting and hiccough; that the latter much aggravated the difficulty. There was intense abdominal pain, with but little tenderness or tympanitis of the bowels. Treatment, morphia and potassa nitras were given internally, and hot fomentation of hops were applied over the abdomen. Inhalation of chloroform gave but transient relief. No food or medicine could be retained upon the stomach, neither did any cathartic act from the beginning. The patient, aged 23 years, had suffered, prior to the attack, with obstinate constipation. The probable exciting cause of his early death was from taking violent exercise at a gymnasium, and bathing, after, in cold walter. He lived from Friday until the following Monday. Autopsy revealed each convolution of the intestines bound, as it were, by lymph. There was three quarts of serous fluid in the peritoneal cavity, but there was no perforation nor fæcal matter in them. Dr. Bogue stated he had seen cases similar, following troumatic injuries, by blows upon the abdomen, rupturing the intestines and the escape of fæcal matter. The symptoms were active peritonitis, and death usually followed within 48 hours. Dr. Gray asked if cold topical applications to the abdomen might be more grateful than warm. Dr. Davis answered that while continuous cold diminishes the amount of blood in the capillaries of the part, and was not always so acceptable, that continuous warmth diminishes the susceptibility and irritability of the part, and he usually made them more soothing by the addition of anodynes; that either application should be uniform in temperature. Dr. Davis reported also a case of death from epilepsy in a lady. She had had paroxysms, two or three per week, lasting a long time. They began after her last confinement. Her memory became much impaired, and there was a peculiar rolling of the eyes; that under the use of belladonna and bromide of ammonium the paroxysms so lengthened that she had but slight symptoms two or three times per week, with divergence of the eyes; that yesterday, while riding out, she became frightened, and this morning her epileptic symptoms returned; that anticipating relief from a bath she repaired to the bath room and locked the door. The servant soon called her but received no answer. She was found dead in the bath tub. The Doctor thinks that after entering the bath she drowned in a paroxysm. Dr. Loverin reported a case of paraplegia in a young man. His eyes were divergent. The commencing cause was syphilis followed by affection of the spinal cord and ramifications of the brain; that he considered the patient liable to die at any time. Dr. Parker reported a case of acute rheumatism: that three days after the attack there were cardiac symptoms; that paroxysms of hiccoughing much aggravated the difficulty. Treatment: he gave morphia and bismuth, chloroform and ether; but more decided relief and cure was found in bromide of ammonium. Dr. Davis believed the locality of the brain affected in the cases reported was the optic thalami; that he had seen the eyes divergent in those cases but the intellect clear; that the disease might finally reach the medulla, affecting respiration, and apoplectic congestion, suffusion and death may supervene. Society adjourned. HIRAM WANZER, *Secretary.*

